# Next Wave of Targets in the Treatment of Advanced Renal Cell Carcinoma

**DOI:** 10.3390/curroncol29080429

**Published:** 2022-07-30

**Authors:** Luisa M. Cardenas, Jasna E. Deluce, Shahrukh Khan, Omar Gulam, Saman Maleki Vareki, Ricardo Fernandes, Aly-Khan A. Lalani

**Affiliations:** 1 Department of Oncology, Juravinski Cancer Centre, McMaster University, Hamilton, ON L8V 5C2, Canada; cardenas@hhsc.ca; 2 London Regional Cancer Program, London Health Sciences Centre, Western University, London, ON N6A 5W9, Canada; jasna.deluce@lhsc.on.ca (J.E.D.); saman.malekivareki@lhsc.on.ca (S.M.V.) ricardo.fernandes@lhsc.on.ca (R.F.); 3 Department of Oncology, Division of Medical Oncology, Schulich School of Medicine & Dentistry, London Health Sciences Centre, Western University, London, ON N6A 5W9, Canada; 4 Faculty of Health Sciences, McMaster University, Hamilton, ON L8S 4L8, Canada; khans197@mcmaster.ca (S.K.); gulamo@mcmaster.ca (O.G.); 5 Division of Experimental Oncology, Department of Oncology, Schulich School of Medicine & Dentistry, Western University, London, ON N6A 5W9, Canada; 6 Department of Pathology and Laboratory Medicine, Schulich School of Medicine & Dentistry, Western University, London, ON N6A 5C1, Canada

**Keywords:** metastatic renal cell carcinoma, immunotherapy, targeted therapy, new targets, HIF2, microbiome, CAR-T, metabolomics

## Abstract

While surgical resection has remained the mainstay of treatment in early-stage renal cell carcinoma (RCC), therapeutic options in the advanced setting have remarkably expanded over the last 20 years. Tyrosine kinase inhibitors targeting the vascular endothelial growth factor receptor (VEGF-TKIs) and anti-programmed cell death 1 (PD-1)/anti-programmed death-ligand 1 (PD-L1)-based immune checkpoint inhibitors (ICIs) have become globally accepted options in the upfront metastatic setting, with different ICI-based combination strategies improving overall survival compared to single-agent Sunitinib. Although some patients benefit from long-term responses, most eventually develop disease progression. Ongoing efforts to better understand the biology of RCC and the different mechanisms of acquired resistance have led to the identification of promising therapeutic targets. Belzutifan, a novel agent targeting the angiogenic pathway involving hypoxia-inducible factors (HIFs), has already been approved for the treatment of early-stage tumors associated with VHL disease and represents a very promising therapy in advanced RCC. Other putative targets include epigenetic regulation enzymes, as well as several metabolites such as adenosine, glutaminase and tryptophan, which are critical players in cancer cell metabolism and in the tumor microenvironment. Different methods of immune regulation are also being investigated, including CAR-T cell therapy and modulation of the gut microbiome, in addition to novel agents targeting the interleukin-2 (IL-2) pathway. This review aims to highlight the emergent novel therapies for RCC and their respective completed and ongoing clinical trials.

## 1. Introduction

Renal cell carcinoma was responsible for over 76,000 new cancer cases and 13,000 deaths in the USA in 2021 [1]. Clear-cell RCC (ccRCC) is the most common subtype and accounts for approximately 70% of cases [2]. Treatment strategies for metastatic renal cell carcinoma (mRCC) have remarkably evolved over the last 20 years, with the vast majority of available scientific evidence focusing on ccRCC. Historically, immunogenic pathways have been important targets in the management of metastatic ccRCC; cytokine therapy with interferon (IFN)-α and interleukin-2 (IL-2) were options for several years, however, their use was limited by significant toxicity and poor response rates [3]. An important feature of ccRCC is its high vascularity nature which is mainly driven by alterations of the VHL tumor suppressor gene, leading to the activation of pro-angiogenic pathways such as the vascular endothelial growth factor (VEGF). The development of small-molecule tyrosine kinase inhibitors (TKIs) targeting VEGF receptors changed the treatment landscape of ccRCC after showing a significantly better efficacy compared to cytokines [4,5]. More recently, the re-emergence of immunotherapy in the form of immune checkpoint inhibitors (ICIs) targeting PD-1/PD-L1 and cytotoxic T-lymphocyte-associated antigen 4 (CTLA-4) receptors once again revolutionized the treatment paradigm of mRCC. Different approaches involving the combination of ICIs or ICI plus TKI therapy have improved survival [6,7,8,9,10]. With the recent change in landscape and the availability of a variety of approved therapeutic options, choosing the proper treatment for the right patient remains an ongoing challenge. While some patients experience long-term responses with ICI-based regimens, others suffer from early disease progression and poorer prognosis. Most patients eventually develop disease resistance and, in an era where both ICIs and TKIs can be used upfront, the optimal sequencing of agents still needs to be elucidated. As our understanding of the biology of RCC and its resistance mechanisms continues to expand, promising novel therapeutic targets are being identified. Here, we present a review of the recent literature in the current treatment landscape of RCC. Furthermore, this review aims to highlight emerging targets in the treatment of mRCC beyond the current ICI landscape and discusses ongoing strategies in managing the heterogeneity of RCC.

## 2. Methods 

A literature search was conducted on PubMed/Medline databases and ASCO library. Key words used for the search included “metastatic renal cell carcinoma”, “immunotherapy”, “checkpoint inhibitors”, “targeted therapy”, “VEGF”, “new targets”, “HIF2a”, “metabolomics”, “CAR-T” and “microbiome.” English-language review articles, clinical trials and guidelines were retrieved. The clinicaltrials.gov database was accessed and the same search terms were used in order to identify ongoing clinical trials of interest. 

## 3. Current First-Line Combination Strategies 

### 3.1. PD-1/CTLA-4 Combination 

Different ICI-based regimens are now FDA- and Health Canada-approved for the upfront treatment of mRCC after showing improved survival in phase III trials. The combination of ipilimumab (anti-CTLA-4 antibody) and nivolumab (anti-PD-1 antibody) was compared to the prior standard of care sunitinib (VEGF-TKI) in the CheckMate 214 trial [6]. Patients with intermediate- or poor-risk disease based on the International Metastatic RCC Database Consortium (IMDC) criteria had a median overall survival (OS) of 47 months with the combination, compared to 26.6 months with sunitinib (HR 0.68; 95% confidence interval (CI) 0.58–0.81); the 5-year survival rates were 43% vs. 31%, respectively. The median duration of response had not been reached at five years with the dual immunotherapy combination compared to 19.7 months with sunitinib [11]. A plateau in progression-free survival (PFS) of 33% was seen after 36 months, suggesting that a proportion of patients are long-term responders [12]. Conditional survival analyses, defined as the probability of remaining alive, progression-free, or in response 2 years beyond the landmark timepoints of 2 and 3 years, support the durability of responses across IMDC subgroups. In the ipilimumab/nivolumab arm, the probability of remaining alive 2 years beyond the 3-year landmark was 81% in the intent-to-treat (ITT) population and 79% in the IMDC intermediate/poor risk group. Although in an exploratory analysis, patients with IMDC favorable-risk did not have a statistically significant benefit with the PD-1/CTLA-4 combination (median OS 74 months vs. 68 months with sunitinib, HR 0.94, CI 0.65–1.37, *p =* 0.7673), the conditional OS in this group was 85% [11]. Treatment-free survival (TFS) has been described as a novel and important outcome that provides information on the quality of survival time. In the CheckMate 214 trial, TFS was found to be higher with the dual immunotherapy combination both in the IMDC intermediate/poor risk group (median TFS 6.9 vs. 3.1 months, 95% CI, 2.5–5.0) and favorable risk group (11 vs. 3.7 months, 95% CI, 4.6–10.0) [13]. 

### 3.2. PD(L)-1/VEGF-TKI Combinations 

Several ICI/TKI combinations are now FDA-approved for the first-line treatment of mRCC. Pembrolizumab (anti-PD-1 antibody) plus axitinib (VEGF-TKI) have become global standards after the combination improved OS compared to sunitinib in the KEYNOTE-426 trial [7]. The primary endpoint was OS in all comers, regardless of IMDC risk score. After a median follow-up of 42 months, the median OS was 45.7 months with pembrolizumab/axitinib vs. 40.1 months with sunitinib (HR 0.73, *p* < 0.001) [14]. Exploratory analysis suggested a lack of an OS benefit in the favorable-risk subgroup (HR 1.06, *p =* 0.58), supporting the hypothesis that the disease biology in this group of patients is different and likely more angiogenesis-driven. Importantly, the combination of pembrolizumab and axitinib resulted in an objective response rate (ORR) of 60% compared to 40% with sunitinib and a complete response (CR) rate of 9% vs. 3% [15]. In the CheckMate 9ER study, nivolumab plus cabozantinib (VEGF-TKI) showed superiority in PFS compared to sunitinib (median PFS 16.6 vs. 8.3 months, HR 0.51, *p* < 0.001) [9] and in OS (median 37.7 months vs. 34.3 months, HR 0.70, 95% CI 0.55–0.90) [16]. The addition of lenvatinib to pembrolizumab, which is thought to synergize the immune response to the PD-1 blockade by decreasing the number of tumor-associated macrophages (TAMs), has shown anti-tumor effects in a number of other malignancies, including advanced endometrial cancer and hepatocellular carcinoma [17]. In the phase III KEYNOTE-577 trial, pembrolizumab plus lenvatinib was associated with a longer PFS and OS in patients with microsatellite stable, advanced endometrial cancer, who had received prior platinum chemotherapy [18]. In advanced hepatocellular carcinoma, the combination has shown an ORR of 46% in a phase Ib trial [19] and it is currently being assessed in the phase III trial LEAP-002 (NCT03713593). In advanced RCC, the combination of pembrolizumab and lenvatinib (VEGF-TKI) was compared to lenvatinib plus everolimus (mTOR inhibitor) and to single-agent sunitinib in the CLEAR study. Pembrolizumab plus lenvatinib showed the longest PFS (median PFS 23.9 months vs. 9.2 with sunitinib, HR 0.39, *p* < 0.001). The probability of survival at 24 months was also the longest with this combination (79% vs. 70% with sunitinib, HR 0.66, *p =* 0.005) [10]. It is important to note that the population in the CLEAR study was characterized by a higher percentage of patients with IMDC favorable-risk disease (27%) and a lower percentage of poor-risk disease (9%) compared to the other ICI-based therapy trials. Lastly, axitinib plus avelumab (anti-PD-L1 antibody) was compared to sunitinib in the JAVELIN Renal 101 trial. Patients with PD-L1-positive tumors had a longer median PFS with the combination (13.8 months vs. 7.2 months, HR 0.61, *p* < 0.001) [8]. Although OS data remain immature, no OS benefit has been demonstrated [20], and other PD(L)-1 plus TKI combinations may, therefore, be preferred. A recent meta-analysis of phase III trials showed that the survival advantage from PD(L)-1/VEGF-TKI combinations over sunitinib was seen regardless of performance status, gender and age [21]. 

Although several ICI-based combinations have shown better and more durable efficacy in the upfront setting compared to single-agent sunitinib, a significant proportion of patients still develop treatment resistance early on. In the CheckMate 214 trial, 20% of patients had progressive disease as their best overall response [6]. The quest for predictive biomarkers that could aid in better patient selection is ongoing [22]. The majority of patients treated with ICI-based combinations eventually develop disease progression. Most guidelines suggest clinical trial participation or single-agent TKIs (with or without mTOR inhibition where indicated) as a subsequent therapy [23,24]. Upfront triplet therapy with anti-PD-1/anti-CTLA-4/TKI is being explored in the COSMIC-313 trial (NCT03937219) and the results of a primary analysis were announced at a recent press release [25]. The combination of nivolumab, ipilimumab and cabozantinib improved PFS compared to the combination of nivolumab and ipilimumab in patients with IMDC-intermediate- or -poor-risk disease (HR 0.73, *p =* 0.01) [25]. The trial is ongoing and the magnitude of benefit from triple therapy will be important to assess the incremental value over doublet combinations. With increasing options for the treatment of advanced RCC, the optimal sequencing of agents remains unknown. Identifying reliable biomarkers of response has been the focus of intensive research. Recent drug development efforts have focused on targeting different biological pathways, including novel angiogenic and immunogenic mechanisms and the tumor microenvironment (TME), with the hope of overcoming ICI and TKI resistance. The composition of the gut microbiome has been associated with response to ICI-based therapy in preclinical and clinical studies and is another target of interest in the treatment of mRCC [26]. While antibiotics may decrease the efficacy of ICIs and have been associated with worse outcomes in patients with mRCC [27,28], the use of proton pump inhibitors (PPIs) does not appear to impact the response to ICIs [29]. Microbiota modulation remains under active investigation as a way of enhancing the efficacy of ICI-based therapies and decreasing their associated toxicities. 

## 4. Novel Targets 

### 4.1. HIF2α Inhibition 

Alterations of the von Hippel–Lindau (VHL) tumor suppressor gene play a critical role in the development of ccRCC. VHL is located on chromosome 3p. Inactivating mutations that affect both alleles are the most common genetic alteration found in ccRCC and these are found both in sporadic and in hereditary forms of ccRCC [30]. VHL disease is the autosomal dominant hereditary cancer syndrome characterized by germline mutations of VHL. Affected individuals have a 70% lifetime risk of developing RCC [31], in addition to multiple other tumor types, including hemangioblastomas and paragangliomas [32]. 

**Figure 1:**
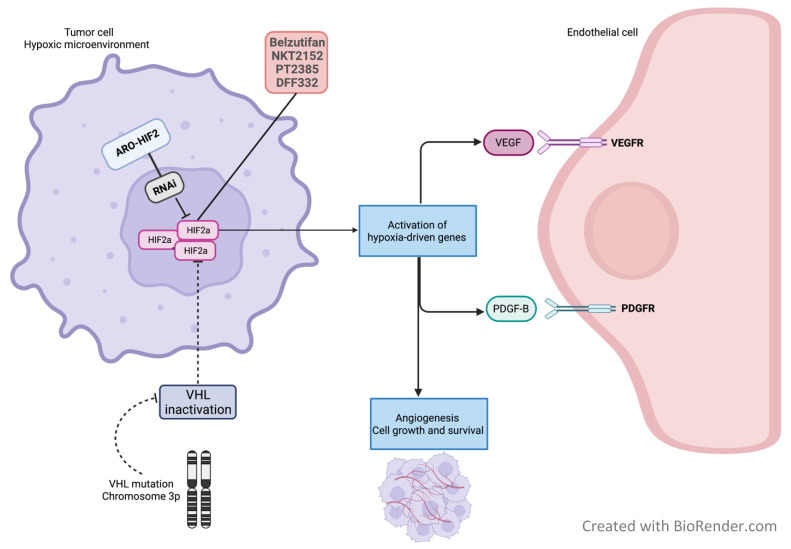
Mechanism of action of HIF2α inhibitors in RCC.

The VHL gene product (pVHL) is a component of an E3 ligase ubiquitin complex, which regulates hypoxia-inducible factors (HIFs) [33]. HIFs are transcription factors comprised of an unstable alpha-subunit and a stable beta-subunit. Of the three isoforms of the alpha subunit (HIF1α, HIF2α and HIF3α), HIF2α has been identified as the key driver of ccRCC [30]. Under normoxic conditions, HIF-1α and HIF-2α are targeted by the pVHL/E3-ubiquitin ligase complex for degradation [34]. Inactivating alterations of VHL result in the accumulation and stabilization of HIFα, which binds to HIFβ. The HIFα/HIFβ heterodimer activates the transcription of downstream hypoxia-driven genes, including VEGF and platelet-derived growth factor-beta (PDGF-β), as well as EPO, which explains the high vascularity and the paraneoplastic erythrocytosis that are often associated with ccRCC [32] (Figure 1). 

In the absence of a known ligand-binding domain, HIF2α had been long considered an undruggable target. However, the discovery of a small pocket that can bind small molecules in the PAS-B domain of HIF2α led to the development of the first-generation HIF2α inhibitor PT2385 [35]. In a phase I dose-escalation trial involving 51 patients with metastatic ccRCC who were heavily pretreated, PT2385 was found to have a favorable safety profile, with anemia, peripheral edema and fatigue being the most common adverse events. Partial responses with PT2385 were seen in 12% of patients, with 2% having a complete response and most (52%) experiencing disease stability [36]. 

Although PT2385 showed a favorable clinical profile, its pharmacokinetics were highly variable, leading to the development of the more selective, second-generation HIF2α inhibitor Belzutifan (MK-6482, previously known as PT2977). This agent was evaluated in a single-arm phase II study, where 61 patients with VHL disease-associated RCC were treated with 120 mg of Belzutifan daily. All patients had localized RCC and pancreatic lesions and 82% had central nervous system (CNS) hemangioblastomas. The ORR in RCC was 49% (95% CI 36 to 62). After a median follow-up of 21.8 months, the median duration of response was not reached. Belzutifan also showed activity in non-RCC neoplasms, with an ORR of 77% in all pancreatic lesions and 91% in the subgroup of patients with pancreatic neuroendocrine tumors. ORR was 30% in CNS hemangioblastomas. The most common toxicities were anemia (any grade in 90%, grade 3 in 8% of patients) and fatigue (any grade in 66%, grade 3 in 5%) [37]. Based on this study, Belzutifan received FDA approval in August 2021 for adults with VHL disease who require therapy for associated RCC and other tumors (CNS hemangioblastomas, pancreatic neuroendocrine tumors), who do not need immediate surgery [38]. 

Belzutifan has also shown activity in the metastatic ccRCC setting. The phase I LITESPARK-001 study showed that in patients with locally advanced or metastatic ccRCC who had received at least one prior therapy, belzutifan monotherapy had an ORR of 25%. After a median follow-up of more than 3 years, the median duration of response was not reached. Anemia was the most common grade 3 adverse event (AE) and was seen in 24% of patients [39]. An ongoing phase II study is evaluating the combination of belzutifan plus cabozantinib in patients with advanced ccRCC who are either treatment-naïve or who have had prior treatment with immunotherapy and/or TKIs (NCT03634540). A preliminary analysis showed that among 53 patients who had received prior therapy, ORR was 22% and 90% had some level of tumor shrinkage. The most common treatment-related adverse events (TRAEs) included anemia (76%), fatigue (68%), hand-foot syndrome (53%) and diarrhea (45%) [40]. Although beyond the objectives of this review, HIF2α inhibition is also being studied in the curative setting. The phase III LITESPARK-022 study is assessing adjuvant belzutifan plus pembrolizumab (NCT05239728), after pembrolizumab monotherapy showed a disease-free survival advantage in this setting [41]. The inhibition of HIF2α via the small molecule NKT2152 in metastatic disease is currently being studied in a phase I/II dose-escalation and expansion trial (NCT05119335). 

RNA interference (RNAi) is a regulatory mechanism in cells that is initiated by double-stranded RNA and leads to gene silencing in a sequence-specific manner. Therefore, RNAi represents a promising therapeutic strategy by the down-regulation of oncogenes, growth factor receptor genes and other signaling molecules involved in carcinogenesis [42]. ARO-HIF2, a therapeutic agent that inhibits the production of HIF2α through RNAi, showed positive interim results in a phase Ib study among patients with metastatic ccRCC (AROHIF21001, NCT04169711). One patient had a partial response in cohort 1 (lower dose, *n* = 7). In cohort 2 (higher dose, *n* = 10), four patients remained on the investigational drug, with stable disease after 12-24 weeks of treatment. The higher dose of 525 mg weekly was well tolerated [43]. Several phase II and III studies evaluating HIF2α inhibitors as single agents or in combination with ICIs and/or TKIs and other similar novel compounds are ongoing (Table 1). 

### 4.2. AXL Inhibition 

AXL is a receptor tyrosine kinase that is normally expressed both in immune and non-immune cells [44]. AXL is upregulated in ccRCC and associated with poor prognosis [45]. AXL activation has been associated with resistance to ICI, by increasing PD-L1 expression and promoting the clearance of tumor antigens, resulting in immune evasion [44]. AXL is essential to the activation of the PI3K/AKT pathway via VEGF and may, therefore, play a role in resistance to antiangiogenic therapies [46]. Batiraxcept is a recombinant fusion protein that inhibits AXL by binding to its activating ligand, GAS6. It is currently being evaluated in a phase 1/2b study in combination with cabozantinib among patients with advanced ccRCC. In an interim analysis, the safety profile was acceptable, with the most common AEs being decreased appetite, diarrhea and fatigue. The ORR was 46%. A baseline ratio of serum soluble AXL (sAXL)/GAS6 of 2.3 or greater was associated with a higher ORR of 67% and may represent a potential biomarker [47]. 

### 4.3. Glutaminase Inhibition

In cancer, hypoxia leads to changes in cellular metabolism that allow the tumor to adapt to a nutrient- and oxygen-restricted microenvironment [48]. In RCC VHL-deficient cells, HIF1α and HIF2α promote metabolic reprogramming towards the use of glutamine. Under hypoxic conditions, this metabolic alteration in tumor cells fuels ATP production via the tricarboxylic acid (TCA) cycle [48]. Glutamine is, therefore, an essential source of energy, carbon and nitrogen, which are crucial for biosynthesis and cellular growth [49]. The first step in glutamine metabolism is the transformation of glutamine into glutamate by the enzyme glutaminase, which is often upregulated in ccRCC [50]. In preclinical models, glutaminase inhibition has suppressed cancer cell growth [51,52,53]. Due to their variable pharmacokinetics and toxicity, the clinical utilization of non-selective glutamine metabolism inhibitors has been limited [50]. Telaglenastat is the first small-molecule, selective glutaminase inhibitor that demonstrated a safe toxicity profile [50]. New evidence suggesting a synergistic effect between telaglenastat and everolimus and cabozantinib has sparked interest in these combination strategies. Everolimus suppresses key glycolytic enzymes by inhibiting the mTOR pathway. Cabozantinib inhibits the growth factor receptors VEGFR, MET and AXL and has downstream effects on the PI3K/AKT/mTOR pathway, leading to a decreased glucose utilization. In a preclinical model, the dual inhibition of glutamine and glucose metabolism showed enhanced anti-tumor activity [54]. 

The combination of telaglenastat plus everolimus was first studied in the ENTRATA trial, a randomized, double-blind, phase II study, where 69 patients with mRCC who had progressed after ≥2 prior systemic therapies were randomized to receive telaglenastat plus everolimus (T + E) vs. placebo plus everolimus (P + E). A trend towards better median PFS was seen with T + E (3.8 months vs. 1.9 months, HR 0.64, *p =* 0.079). Grade ≥3 adverse events (AEs) occurred in 80% (T + E) vs. 60% (P + E), with the most common being anemia (17% vs. 17%), pneumonia (7% vs. 4%), abdominal pain (7% vs. 0%), thrombocytopenia (7% vs. 0%) and fatigue (4% vs. 9%). Discontinuation due to AEs occurred in 28% (T + E) vs. 30% (P + E) [55]. The phase II CANTATA trial was a randomized, placebo-controlled, double-blind study that subsequently evaluated the combination of telaglenastat plus cabozantinib (T + C) vs. placebo plus cabozantinib (P + C) in patients with advanced ccRCC who had progressed on prior first- or second-line therapy, including anti-angiogenic or ICI-based regimens. Among the 444 randomized patients, there was no statistically significant difference in PFS (median PFS 9.2 months with T + C vs. 9.3 months with P + C, HR 0.94, *p =* 0.65). ORR was similar between both groups (31% vs. 28%, respectively), and the OS data were immature at the data cutoff. In a prespecified subgroup analysis, there was a trend towards PFS benefit with T + C in patients who had received prior ICI-based therapy (11.1 vs. 9.2 months, HR 0.77). Grade 3–4 AEs occurred in 71% of patients on T + C vs. 79% in those on P + C. The most common grade 3–4 AEs were hypertension (17% vs. 18%) and diarrhea (15% vs. 13%) [56]. 

### 4.4. Adenosine Receptor Inhibition

Metabolites play an essential role in modulating immune responses and, through metabolic alterations, are involved in resistance mechanisms to ICIs [57]. In addition to metabolites representing potential therapeutic strategies, metabolomics is becoming increasingly important for patient stratification and monitoring during clinical trials [58]. The breakdown of extracellular ATP into immunosuppressive adenosine is a known escape mechanism from anti-tumor immunity [59]. ATP is an abundant metabolite that is released in the extracellular space following pro-inflammatory stimuli. It promotes immune responses by mediating the production of cytokines and activation of T cells [60]. Immune regulatory mechanisms are in place to protect tissues from excessive immune reactions. One of these mechanisms occurs by converting ATP into adenosine by two ectonucleotidases: CD39 and CD73 [60]. Adenosine exerts its immunosuppressive effects through the adenosine receptors A2A and A2B, which are expressed on different subsets of immune cells [61]. In the TME, hypoxic conditions lead to the overexpression of CD39 and CD73, stimulating the breakdown of ATP into adenosine and accumulating it in high concentrations [62]. Additionally, hypoxia upregulates the expression of A2A and A2B, increasing cell responsiveness to adenosine and favoring immune evasion [60]. Compared to other solid tumors, RCC has been found to have higher levels of expression of A2AR and CD73 [63]. A phase I clinical trial evaluated the small molecule A2AR antagonist, ciforadenant (previously known as CPI-444), in patients with advanced refractory RCC, showing clinical responses both as monotherapy and in combination with atezolizumab (anti-PD-L1 antibody). The expression of higher baseline levels of adenosine-induced genes in tumor biopsies was associated with tumor regression and represents a potential predictive biomarker to identify patients who are more likely to respond to the inhibition of the adenosine pathway [63]. 

### 4.5. Tryptophan Catabolism Pathway 

Indoleamine 2, 3-dioxygenases 1 (IDO1) is a catabolic enzyme that catabolizes tryptophan, causing immunosuppression in the TME. The depletion of tryptophan results in the activation of regulatory T cells and myeloid-derived suppressor cells, promoting immune tolerance [64]. Epacadostat, a potent IDO1 inhibitor, decreases the metabolism of tryptophan, which ultimately promotes immunosurveillance by increasing the proliferation of effector T cells and natural killer cells and decreasing the expansion of regulatory T cells [65]. Epacadostat plus pembrolizumab was evaluated in a phase I trial among patients with advanced solid tumors. Among the 33 patients in the advanced RCC cohort, ORR was 47% in patients with zero-to-one prior treatments and 37% in patients who had two or more prior therapies. The most common TRAEs included fatigue, rash, arthralgia and diarrhea [66]. The combination is currently under investigation as a first-line therapy in a phase III trial, compared to the previous standard of care (sunitinib or pazopanib) (NCT03260894). 

### 4.6. Histone Deacetylase Pathway 

Histone deacetylases (HDACs) are key enzymes involved in epigenetic regulation. HDACs promote histone deacetylation, resulting in chromatin condensation and the suppression of gene transcription [67]. HDACs are key components in HIF-1α transcriptional activity and signaling [68], and are thought to be involved in the resistance to anti-angiogenic therapies [69]. HDACs have been shown to be upregulated in RCC. The inhibition of HDACs results in lower cell proliferation and the activation of apoptosis [70], as well as the downregulation of HIF-1α expression in hypoxic conditions [69]. Abexinostat is an HDAC inhibitor that, in combination with pazopanib, showed anti-tumor activity and acceptable toxicity in a phase I trial [69]. Among patients with advanced RCC (*n*= 22), 7/10 patients who had prior disease progression with single-agent pazopanib achieved a reduction in tumor burden with the combination. The median duration of response was 9.1 months. Five patients who were treatment-refractory achieved durable partial-tumor responses lasting longer than 2 years [71]. A randomized phase III trial, RENAVIV, is underway comparing pazopanib plus abexinostat vs. pazopanib plus placebo in the first- or second-line settings in patients with advanced RCC (NCT03592472). 

### 4.7. Novel Immunotherapy Pathways 

#### 4.7.1. IL-2 Pathway

RCC is considered an immunogenic cancer; therefore, modulating the immune system’s anti-tumor response has been a therapeutic target of interest in RCC for several years. One of the historical standards is high-dose IL-2, which was first introduced in the mid-80s [72]. However, given a poor ORR close to 20% and a highly toxic profile [3], its use has been replaced mainly by contemporary targeted therapies and, more recently, ICIs. Although some individuals benefit from long-term responses with ICIs, resistance to these agents ultimately occurs in most patients. Some of the factors associated with resistance are low levels of tumor PD-L1 expression and of baseline tumor-infiltrating lymphocytes, as well as T cell exhaustion in the TME [73]. 

Nemvaleukin (ALKS 4230), a novel IL-2 receptor agonist, is a fusion protein that interacts with the intermediate-affinity IL-2 receptor, also selectively activating effector T cells in the TME without stimulating regulatory T cells [74]. In the ARTISTRY-1 phase I/II study, ALKS 4230 was evaluated as monotherapy and in combination with pembrolizumab in patients with refractory solid tumors. Among 16 evaluable patients with RCC, one patient achieved a partial response with monotherapy. The toxicity profile was found to be acceptable, with the most common TRAEs including chills and pyrexia [75]. The combination phase of this trial is ongoing (NCT03861793). NKTR-214 is another novel prodrug targeting the IL-2 receptor, promoting the proliferation and activation of CD8^+^ and natural killer (NK) T cells in the TME. In the phase III trial PIVOT-09 (NCT03729245), bempegaldesleukin (NKTR-214) was evaluated in combination with nivolumab vs. either sunitinib or cabozantinib in the upfront metastatic ccRCC setting. Preplanned analyses showed that the combination did not meet the prespecified boundary for statistical significance for ORR and OS and the trial was terminated early [76]. 

#### 4.7.2. CAR-T Cell Therapy 

Chimeric antigen receptor (CAR)-T cell therapy is a type of adoptive immunotherapy that has revolutionized the treatment of hematological malignancies and is under active investigation in solid tumors. T-cells play a critical role in anti-tumor immunity and immune memory against tumors. Despite RCC tumors being highly infiltrated by T cells, they do not always exert effective anti-tumor responses, potentially due to the concomitant presence of regulatory T cells and myeloid cells and other immunosuppressive mechanisms [77]. CAR-T cell therapy represents a potential strategy to restore T-cell anti-tumor activity through a bioengineering process that results in the expression of a chimeric antigen receptor (CAR) on T cells. CARs interact with their target antigens on tumor cells with high specificity and they are not restricted by a major histocompatibility complex (MHC), allowing them to be active in tumors with low levels of MHC expression [78]. Several challenges of CAR-T cell therapy in solid tumors have been identified, including a hostile TME leading to the elimination of CAR-T cells, as well as the lack of receptor specificity [78]. Furthermore, potentially severe side effects due to off-target, on-tumor toxicities represent a critical limiting factor in implementing CAR-T cell therapy for solid tumors. Cytokine release syndrome, macrophage activation syndrome and acute kidney injury are some of the TRAEs reported in hematologic malignancies and are particularly relevant in patients with mRCC who often have prior nephrectomy and/or concomitant decreased renal function [78]. 

A first-generation CAR directed against carboxy-anhydrase-IX (CAIX), a highly expressed enzyme in RCC, was studied in a phase I/II trial, where 12 patients with previously treated mRCC received a maximum of 10 daily CAR-T infusions. Circulating CAR T-cells were detectable transiently in all patients. The treatment had to be stopped in half of the participants due to the development of grade 2–4 liver enzyme abnormalities. Unfortunately, no therapeutic effect was found [79]. The combination of a second-generation CAR targeting CAIX plus sunitinib showed synergistic effects in animal models of mRCC. Such a combination strategy can take advantage of the immunomodulatory effects of sunitinib, rendering adoptive immunotherapy potentially more effective [80]. There are currently several ongoing studies of CAR-T cell therapy in mRCC against different targets, including CAIX (NCT04969354), CD70 (COBALT-RCC, NCT04438083), and AXL/ROR2 (NCT03393936). 

### 4.8. Microbiome 

Several mechanisms have been implicated in cancer evasion from the immune system, including an increased expression of immune checkpoints, downregulation of surface antigens on cancer cells, recruitment of immunosuppressive cells, such as regulatory T cells and myeloid-derived suppressor cells, and secretion of toxic metabolites [81]. The gut microbiome has been recognized as a central component of immune cancer surveillance [82]. Commensal gut bacteria can influence local and systemic immune responses through numerous complex pathways, including the synthesis or transformation of circulating metabolites, phagocyte activation and the production of short-chain fatty acids (SCFAs) that impact proliferation and cell death [26,83]. The modulation of the gut microbiome to augment the efficacy of ICI-based therapy is an emerging therapeutic strategy. In a preclinical study, the oral administration of *Bifidobacterium* to mice with melanoma enhanced the maturation of dendritic cells and stimulated CD8^+^ T cell accumulation in the TME, restoring the efficacy of anti-PD-L1 therapy [84]. Another study suggested that antibiotic use decreases the effect of anti-CTLA-4 therapy in mice. The administration of *Bacteroides fragilis* enhanced T helper cell activation and maturation of intratumoral dendritic cells, enhancing the efficacy of CTLA-4 blockade [85]. Since the publication of these hallmark preclinical studies, a number of human studies have shown that the composition and diversity of the gut microbiome influence the response to ICIs in several cancers, including melanoma, non-small cell lung cancer and RCC [26]. 

In patients with mRCC, baseline stool samples taken before starting ICI-based therapy were compared to samples taken after 12 weeks of therapy. Several species were associated with a clinical benefit or lack thereof [86]. Antibiotic use has been shown to alter the composition of the gut microbiome [87], in addition to being associated with a lower clinical response and shorter PFS in patients receiving ICI-based therapy [27]. Modulation of the gut microbiome to improve outcomes in patients with mRCC is currently under investigation. In a phase I trial, patients were randomized to receive first-line ipilimumab plus nivolumab with or without CBM-588, a strain of *Clostridium butyricum* that is commonly used as a probiotic in Japan to prevent antibiotic-induced diarrhea [88]. Patients who took CBM-588 had temporal changes in their gut microbiome composition, with a higher increase of *B. adolescentis* and *C. butyricum* compared to baseline. Additionally, *C. butyricum* was only detected in patients receiving CBM-588 and pathogenic species such as *E. coli* and *Klebsiella spp.* were detected more frequently in patients not receiving the investigational agent. Clinical response to ICI-based therapy was improved among patients receiving CBM-588, with higher ORR (59% vs. 11%, *p =* 0.024) and longer mPFS (NR vs. 11 weeks, *p* < 0.001) [89]. The gut microbiome has also been associated with toxicity to ICI-based therapy. This is particularly important in patients who receive double immune checkpoint blockades with anti-CTLA4 and anti-PD-1 antibodies, where the rate of grade ≥3 immune-related adverse events (irAEs) can be as high as 60% [90]. In patients with melanoma treated with ipilimumab plus nivolumab, certain bacterial taxa were more frequently detected in baseline stool samples of those who experienced grade ≥3 irAEs, including *B. intestinalis* and *I. bartlettii* [91]. The PERFORM clinical trial is currently investigating whether fecal microbiota transplantation (FMT) from healthy donors can decrease the rate of irAEs in mRCC patients treated with immunotherapy-based combinations (NCT04163289). Microbiome signatures may become future biomarkers, not only for ICI efficacy, but also for safety.

In addition to probiotics and FMT, a number of other strategies to modulate the gut microbiome and enhance response to ICI in different cancers remain under investigation in early phase trials. The development of engineered microbiomes provides an opportunity to attenuate bacterial virulence, while taking advantage of the anti-tumor activity induced by specific bacterial strains, such as Escherichia coli, Bifidobacterium, Clostridium and Salmonella typhimurium [92]. Through engineered microbial therapies, the modulation of metabolites in the TME can also enhance the response to ICIs [26]. Other interesting microbiome-modulation strategies that are being studied in early phase clinical trials include tumor-therapeutic vaccines and bacteriophages [26]. The administration of the oral short-chain fatty acid valproic acid, which is a microbial metabolite, is also being investigated in several malignancies (NCT02624128, NCT01106872). Another area of interest is the modulation of the gut microbiome through dietary modification. A calorie-restricted ketogenic diet, in addition to a glutamine antagonist in mice with glioblastoma resulted in the killing of tumor cells, reversal of disease-related symptoms and improved survival [93]. 

Several limitations remain in the study of ICIs and microbiota. Positive results in preclinical studies do not always translate to anti-tumor efficacy in humans, highlighting the complexity of the interaction between the gut microbiome, the host’s immune system and the TME [83]. A deeper understanding of the immunosuppressive and immunostimulatory effects of specific bacterial strains and their metabolites is needed. Additionally, heterogeneity in the methods of RNA sequencing analysis can lead to challenges generalizing results and needs to be taken into consideration when interpreting studies [83].

## 5. Conclusions

Remarkable progress has been made in the treatment of advanced RCC over the last 20 years, with ICI-based combination strategies in the upfront setting improving overall survival. Ongoing challenges, including acquired resistance and the prevalence of irAEs, continue to motivate efforts to discover new therapeutic targets. Current research focuses on identifying biomarkers that may serve as therapeutic targets or predictors of therapeutic responses. Promising pathways under active investigation include angiogenic mechanisms through HIFs, immune modulation through CAR-T cell therapy, the gut microbiome and critical molecules that affect cellular metabolism, immunosurveillance and the TME. Some of these targets are already either FDA-approved in special settings or being studied in phase II and III clinical trials and may once again revolutionize the treatment of mRCC. As our therapeutic tools expand, identifying patients who are more likely to benefit from specific therapies with a low risk of AEs will be critical. Advances in the understanding of predictive biomarkers will hopefully result in the delivery of more personalized cancer care and continue to improve the outcomes of patients with RCC.

## Figures and Tables

**Table 1:** Summary of active phase II and III trials evaluating novel therapeutic targets in RCC.

Target	Clinical Trial	Phase	Population	Treatment Arm	Control Arm	Primary Endpoint
HIF2α						
	NCT03108066	II	VHL-disease-associated ccRCC (early-stage)	PT2385	N/A	ORR
	MK-6482-003 (NCT03634540)	II	Advanced ccRCC	Cohort 1: Belzutifan + Cabozantinib (treatment naïve) Cohort 2: Belzutifan + Cabozantinib (prior immunotherapy)	N/A	ORR
	MK-6482-013 (NCT04489771)	II	Advanced RCC with clear cell component, prior PD(L)-1	Belzutifan	N/A	ORR
	MK-6482-005 (NCT04195750)	III	Advanced ccRCC after prior PD(L)-1 and VEGF-targeted therapy	Belzutifan	Everolimus	PFS OS
	MK-6482-012 (NCT04736706)	III	Advanced, untreated ccRCC	A: Pembrolizumab + Belzutifan + Lenvatinib B: Pembrolizumab/Quavonlimab + Lenvatinib	Pembrolizumab + Lenvatinib	PFS OS
	MK-6482-011 (NCT04586231)	III	Advanced RCC with clear cell component, prior PD(L)-1	Belzutifan + Lenvatinib	Cabozantinib	PFS OS
	LITESPARK-022 (NCT05239728)	III	Clear cell RCC post-curative-intent nephrectomy	Belzutifan + Pembrolizumab	Placebo + Pembrolizumab	DFS
IL-2						
	NCT03991130	II	Advanced RCC, prior PD(L)-1	High dose IL-2 + Nivolumab	N/A	ORR
	NCT02306954	II	Advanced ccRCC	High dose IL-2 + SBRT to metastatic foci	High dose IL-2	ORR
	NCT01884961	II	Advanced RCC	High dose IL-2 + boost of radiotherapy to metastatic foci	N/A	Immunological efficacy Predictive biomarkers
	NCT03501381	II	Advanced ccRCC	High dose IL-2 + Entinostat	High dose IL-2	PFS
	NCT02964078	II	Advanced RCC, clear cell component	IL-2 + Pembrolizumab	N/A	ORR
Tryptophan catabolism	NCT03260894	III	Advanced RCC, clear cell component	Epacadostat + Pembrolizumab	Sunitinib or Pazopanib	ORR
HDAC	RENAVIV (NCT03592472)	III	Advanced RCC, clear cell component	Pazopanib + Abexinostat	Pazopanib + Placebo	PFS
